# Generalized enhanced suffix array construction in external memory

**DOI:** 10.1186/s13015-017-0117-9

**Published:** 2017-12-07

**Authors:** Felipe A. Louza, Guilherme P. Telles, Steve Hoffmann, Cristina D. A. Ciferri

**Affiliations:** 1 Department of Computing and Mathematics, University of São Paulo, Av. Bandeirantes, 3900, Ribeirão Preto, 14040-901 Brazil; 2 Institute of Computing, University of Campinas, Av. Albert Einstein, 1251, Campinas, 13083-852 Brazil; 3 Computational Biology, Leibniz Institute on Aging - Fritz Lipman Institute and Friedrich Schiller University Jena, Beutenbergstrasse 11, Jena, 07745 Germany; 4 Institute of Mathematics and Computer Science, University of São Paulo, Av. Trabalhador São-carlense, 400, São Carlos, 13560-970 Brazil

**Keywords:** Suffix array, LCP array, Burrows–Wheeler transform, External memory algorithms, String collections

## Abstract

**Background:**

Suffix arrays, augmented by additional data structures, allow solving efficiently many string processing problems. The external memory construction of the generalized suffix array for a string collection is a fundamental task when the size of the input collection or the data structure exceeds the available internal memory.

**Results:**

In this article we present and analyze $\mathsf {eGSA}$ [introduced in CPM (External memory generalized suffix and $\mathsf {LCP}$ arrays construction. In: Proceedings of CPM. pp 201–10, 2013)], the first external memory algorithm to construct generalized suffix arrays augmented with the longest common prefix array for a string collection. Our algorithm relies on a combination of buffers, induced sorting and a heap to avoid direct string comparisons. We performed experiments that covered different aspects of our algorithm, including running time, efficiency, external memory access, internal phases and the influence of different optimization strategies. On real datasets of size up to 24 GB and using 2 GB of internal memory, $\mathsf {eGSA}$ showed a competitive performance when compared to $\mathsf {eSAIS}$ and $\mathsf {SAscan}$, which are efficient algorithms for a single string according to the related literature. We also show the effect of disk caching managed by the operating system on our algorithm.

**Conclusions:**

The proposed algorithm was validated through performance tests using real datasets from different domains, in various combinations, and showed a competitive performance. Our algorithm can also construct the generalized Burrows-Wheeler transform of a string collection with no additional cost except by the output time.

## Introduction

Suffix arrays [40] (also known as PAT arrays [23]) may be used for the solution of string processing problems in several areas, including pattern matching, data compression and information retrieval [24, 39, 47]. Combining a suffix array with the longest common prefix ($\mathsf {LCP}$) array and with the Burrows–Wheeler transform ($\mathsf {BWT}$) [12] provides a data structure, an enhanced suffix array (ESA) [2], that enables solving many string processing problems in optimal time and space.

Using such structures in the solution of problems involving strings is usually done in two steps: the structure is first constructed and then it is queried. This article is about the construction of generalized enhanced suffix arrays for a collection of strings using external memory. This is motivated by the rising number of applications that deal with huge sets of strings, such as those in Bioinformatics and Internet searching. Moreover, recent advancements in non-volatile storage technologies have substantially narrowed the gap between internal and external memory access times, making the querying of external suffix arrays significantly faster.

Different algorithms have been proposed for internal memory suffix array construction (see [17, 49]), including algorithms with linear running time [30, 33, 46]. Gonnet et al.  [23] proposed the first external memory algorithm for constructing suffix arrays. Later, Crauser and Ferragina [14] adapted internal memory algorithms to work in external memory. Dementiev et al.  [16] observed that these algorithms do not scale well and presented a pipelined version of the internal memory algorithm DC3 [30] to external memory. Nong et al.  [44, 45] adapted the internal memory algorithms SA-DS and SA-IS [46] to external memory, and Liu et al.  [34] presented an enhanced version of SA-IS to external memory. Kärkkäinen and Kempa [25] presented the $\mathsf {SAscan}$ algorithm, improving on the earlier proposal by Gonnet et al.  [23], and later, Kärkkäinen et al.  presented a parallel external version of $\mathsf {SAscan}$ algorithm [27].

$\mathsf {BWT}$ can be either obtained from the suffix array or constructed directly in internal memory in linear time [48]. Ferragina et al.  [18] proposed an external memory algorithm to construct the $\mathsf {BWT}$ for a single string, and Bauer et al.  [5] presented external memory algorithms to compute and decode the $\mathsf {BWT}$ for a string collection.

$\mathsf {LCP}$ construction in internal memory is also possible in linear time during the suffix array construction [19, 35] or afterwards, given the suffix array [29, 31, 41] or the $\mathsf {BWT}$ as input [7, 22]. Kärkkäinen and Kempa [26] presented the $\mathsf {LCPscan}$, an external memory algorithm to construct $\mathsf {LCP}$ arrays given the suffix array as input, and Bauer et al.  [6] proposed the $\mathsf {extLCP}$ algorithm to construct both $\mathsf {BWT}$ and $\mathsf {LCP}$ arrays for large collections of equally sized strings in external memory, and later, Cox et al.  [13] presented an extended version of $\mathsf {extLCP}$ to deal with strings with different sizes.

The suffix and the $\mathsf {LCP}$ arrays are constructed together in external memory by $\mathsf {eSAIS},$ proposed by Bingmann et al. [10], one of the most efficient external memory algorithm to date. There exists alternatives to compute suffix and $\mathsf {LCP}$ arrays in parallel [20] and using small space [37, 42].

In this article we present and analyze the algorithm $\mathsf {eGSA}$ (introduced in [38]) in depth. To our knowledge this is the first algorithm to construct generalized enhanced suffix arrays in external memory. We compared $\mathsf {eGSA}$ with the most efficient related algorithms in the literature, $\mathsf {eSAIS}$ [10] and $\mathsf {SAscan}$ [25]. Although $\mathsf {eSAIS}$ and $\mathsf {SAscan}$ can easily be applied to the concatenation of a string collection, our method is shown to run faster in practice. In addition to the $\mathsf {LCP}$ array, our method also constructs the $\mathsf {BWT}$ for the collection. $\mathsf {eGSA}$ uses a heap and a combination of optimization procedures that are shown to be very effective in practice. The optimizing strategies that we propose in this article are based on nice properties of strings and their relation with the $\mathsf {LCP}$ array, and are applied across the nodes of a heap.

Theoreticallly, $\mathsf {eSAIS}$ runs in $O(n \log _{M/B}(n/B))$ time and $O((n/B) log_{M/B}(n/B))$ I/Os, where *n* is the length of the input string, *B* is the disk block size and *M* is the RAM size. $\mathsf {SAscan}$ runs in $O((n^2/M) \log (2+\log _{\sigma }/\log \log n))$ time and $O(n^2\log \sigma /(MB\log n) + (n/B) \log _{M/B} (n/B))$ I/Os. Our algorithm runs in $O((N \log m) { maxlcp})$ time and $O(N \log m |T_\ell |)$ I/Os, where *N* is the sum of the *m* string lengths in the input, ${ maxlcp}$ is the length of the longest common prefix between suffixes of the input strings, $|T_\ell |$ is the length of the longest string in the collection.

The rest of the article is organized as follows.  "Background" section introduces concepts and notation, "eGSA" section describes the algorithm and presents a theoretical analysis, "Performance evaluation" section details the experiments, results and investigates limitations of the algorithm. "Conclusions" section concludes the article.

## Background

Let $\Sigma$ be an ordered alphabet of symbols. We denote the set of every string of symbols in $\Sigma$ by $\Sigma ^*$ and the concatenation of strings or symbols by the dot operator ($\cdot$). Let $\$$ be a symbol not in $\Sigma$ that precedes every symbol in $\Sigma$ with respect to the alphabetical order. We define $\Sigma ^{\$} = \{T\cdot \$ | T \in \Sigma ^* \}.$ We use the symbol < for the lexicographic order relation between strings.

The *i*th symbol in a string *T* of length *n* is denoted *T*[*i*], $1\le i\le n.$ A substring of *T* is denoted $T[i,j] = T[i]\cdot \ldots \cdot T[j],$ $1\le i\le j \le n.$ A prefix of *T* is a substring of the form *T*[1, *k*] and a suffix is a substring of the form *T*[*k*, *n*], $1\le k\le n.$

A suffix array for a string $T\in \Sigma ^{\$}$ of size *n*, denoted $\mathsf {SA},$ is an array of integers $\mathsf {SA} =[i_1, i_2, \ldots , i_n]$ such that $T[i_1,n]<T[i_2,n]<\cdots <T[i_n,n].$ Thus, a suffix array provides the lexicographic order for all suffixes of a string.

Let ${ pos} (T[k,n])$ denote the mapping of suffix *T*[*k*, *n*] to its position in $\mathsf {SA},$ i.e. the reverse suffix array, and let ${ suff} (j)$ denote the mapping of position *j* of $\mathsf {SA}$ to the suffix represented at *j*, namely $T[\mathsf {SA} [j],n]$.

Let ${ lcp} (S,T)$ be the length of the longest common prefix of two strings *S* and *T* in $\Sigma ^{\$}.$ The $\mathsf {LCP}$ array for *T* is an array of integers such that $\mathsf {LCP} [i] = { lcp} (T[\mathsf {SA} [i],n],T[\mathsf {SA} [i-1],n])$ and $\mathsf {LCP} [1] = 0$.

The $\mathsf {BWT}$ is a reversible transformation obtained through cyclic rotations of a string, and results in another string that is easier to compress [12]. The $\mathsf {BWT}$ has a close relationship to the suffix array and can be trivially obtained from it. Let the $\mathsf {BWT}$ of a string *T* be denoted $\mathsf {BWT}$ and defined as $\mathsf {BWT} [i]=T[\mathsf {SA} [i]-1]$ if $\mathsf {SA} [i]\ne 1$ or $\mathsf {BWT} [i]= \$$ otherwise.

We will refer to the structure formed by $\mathsf {SA},$ $\mathsf {LCP},$ $\mathsf {BWT}$ as an enhanced suffix array, denoted $\mathsf {ESA}$ [2]. Table 1 shows the enhanced suffix array for $T_1={ GATAGA\$}$ and for $T_2={ TAGAGA\$}.$

**Table 1:**
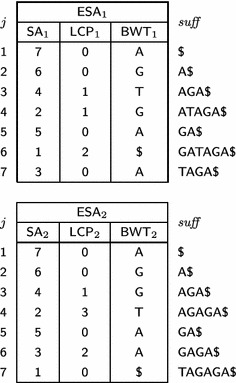
Enhanced suffix arrays for $T_1 = GATAGA\$$ and for $T_2 = TAGAGA\$$

Let $\mathcal {T}$ be a collection of *m* strings $\{T_1,\ldots ,T_m\}$ from $\Sigma ^{\$}$ having lengths $n_1,\ldots ,n_m.$ We extend the lexicographic relation among strings to deal with unit length suffixes of $\mathcal {T}:$ let < be augmented for pairs of suffixes of length 1 of strings in $\mathcal {T}$ by $T_i[n_i,n_i] < T_j[n_j,n_j]$ if $i<j.$

The generalized suffix array of $\mathcal {T},$ denoted $\mathsf {GSA},$ is an array of pairs of integers (*a*, *b*) that specifies the lexicographic order of all suffixes $T_a[b,n_a]$ of strings in $\mathcal {T}.$ We denote the first component of $\mathsf {GSA} [j]$ as $\mathsf {GSA} [j].{ str} \in [1,m]$ and the second as $\mathsf {GSA} [j].{ suf} \in [1, \max \{n_1,\ldots ,n_m\}].$ Also, we extend the function ${ suff} (j)$ to map the suffix represented at position *j* of $\mathsf {GSA},$ namely $T_{\mathsf {GSA} [j].{ str}}[\mathsf {GSA} [j].{ suf}, n_{\mathsf {GSA} [j].{ str}}].$

The generalized $\mathsf {LCP}$ of $\mathcal {T}$ is defined as $\mathsf {LCP} [j] = { lcp} ({ suff} (j), { suff} (j-1))$ and $\mathsf {LCP} [1] = 0,$ and the generalized $\mathsf {BWT}$ of $\mathcal {T}$ is defined as $\mathsf {BWT} [j]=T_{\mathsf {GSA} [j].{ str}}[\mathsf {GSA} [j].{ suf}-1]$ if $\mathsf {GSA} [j].{ suf} \ne 1$ or $\mathsf {BWT} [j]=\$$ otherwise.

The generalized suffix array of $\mathcal {T}$ together with its corresponding $\mathsf {LCP}$ array and $\mathsf {BWT}$ will be called generalized enhanced suffix array and denoted $\mathsf {GESA}.$ Table 2 shows the generalized enhanced suffix array for $\mathcal {T}= \{T_1, T_2\},$ where $T_1 = { GATAGA\$}$ and $T_2 = { TAGAGA\$}.$

**Table 2:**
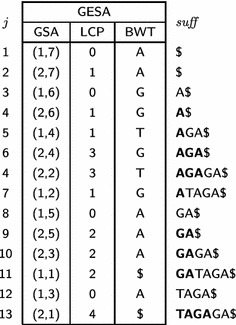
Generalized enhanced suffix array for $\mathcal {T}= \{GATAGA\$, TAGAGA\$\}$

The ${ suff}$ column illustrates, in bold, prefixes shared between consecutive positions in the array

## eGSA

The External Generalized Enhanced Suffix Array Construction Algorithm ($\mathsf {eGSA}$) resembles a two-phase multiway merge-sort [32]. Algorithm 1 illustrates $\mathsf {eGSA}$ without the otimizing strategies introduced in Phase 2. Phase 1 builds the enhanced suffix arrays for the input strings and Phase 2 merges the respective arrays using an improved string comparison method on memory buffers. We detail each phase below.
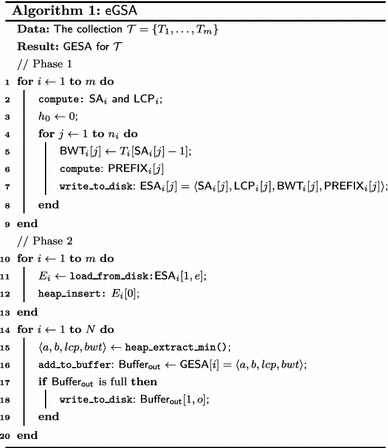


### Phase 1: internal sorting

The input for $\mathsf {eGSA}$ is a collection $\mathcal {T}$ of *m* strings $\mathcal {T}=\{T_1,\ldots ,T_m\}$ having lengths $n_1,\ldots ,n_m$ with total length *N* and stored in external memory.

In Phase 1 the suffix array $\mathsf {SA} _i$, the $\mathsf {LCP}$ array $\mathsf {LCP} _i ,$ the Burrows–Wheeler transform $\mathsf {BWT} _i$ and the auxiliary array $\mathsf {PREFIX} _i$ are built for each $T_i$ and stored in external memory (lines 1–9 of Algorithm 1). Any internal or external memory suffix and $\mathsf {LCP}$ array construction algorithm may be used by $\mathsf {eGSA}$ to build $\mathsf {SA} _i$ and $\mathsf {LCP} _i$ (line 2). As they are constructed, both $\mathsf {BWT} _i$ (line 5) and $\mathsf {PREFIX} _i$ (lines 6) can be computed and written sequentially to external memory with no need to store in internal memory.

$\mathsf {PREFIX}$ arrays are used to reduce external memory accesses in Phase 2: starting from a position *j* and concatenating $\mathsf {PREFIX} _i$ successively for adjacent preceding positions will render a prefix of $T_i[j,n_i]$, up to a position with ${ lcp}$ equal to zero. In other words, $\mathsf {PREFIX} _i [j]$ will store symbols from the string, such that the contents of $\mathsf {PREFIX} _i [j]$ concatenated to parts of preceding positions of $\mathsf {PREFIX}$ is equal to a starting portion of the suffix at position $\mathsf {SA} _i [j].$ More formally, let *p* be a given integer constant. Let $h_0=0$ and $h_{j}=\min (\mathsf {LCP} _i [j],h_{j-1}+p).$ We define $\mathsf {PREFIX} _i [j]=T_i[\mathsf {SA} _i [j]+h_{j},\mathsf {SA} _i [j]+h_{j}+p].$ As a boundary condition, whenever the length of $T_i$ is exceeded, sufficient $\$$ symbols are added to the right of $\mathsf {PREFIX} _i [j].$ An example for the $\mathsf {ESA}s$ from Table 1 with $p = 3$ is shown in Table 3. Notice that it is possible to recall the strings with the aid of $\mathsf {PREFIX}.$ Our construction is similar to the left-justified approach by Sinha et al.  [50] and relates to the work of Barsky et al.  [4].

**Table 3:**
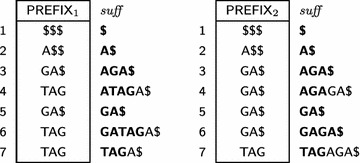
Prefix array examples

The ${ suff}$ column illustrates, in bold, the prefixes recovered without external access during the merging phase of our algorithm, as detailed in "Phase 2: external merging" section

We will denote a tuple of elements in the same position of an $\mathsf {ESA}$ augmented with the $\mathsf {PREFIX}$ array by $\mathsf {ESA} _i[j]=\langle \mathsf {SA} _i [j],\mathsf {LCP} _i [j],\mathsf {BWT} _i [j],\mathsf {PREFIX} _i [j]\rangle,$ and we will use a dot to refer to a component, for instance $\mathsf {ESA} _i[j].\mathsf {SA} _i.$ The product of Phase 1 is $\mathsf {ESA} _1, \ldots , \mathsf {ESA} _m.$

### Phase 2: external merging

Phase 2 merges the enhanced suffix arrays computed in Phase 1 to obtain a $\mathsf {GESA}$ for $\mathcal {T}.$

Each $\mathsf {ESA}$ is partitioned into consecutive blocks having *e* consecutive elements, except perhaps for the last block. For each $\mathsf {ESA} _i$ the algorithm uses two internal memory buffers: a string buffer $S_i,$ with capacity for at most *s* symbols of $T_i,$ and an enhanced-array buffer $E_i,$ large enough to store a block of $\mathsf {ESA} _i.$ It also uses two other buffers: an output buffer $\mathsf {Buffer_{out}}$ for at most *o* elements of the $\mathsf {GESA},$ and an induced buffer *I*, of size $|\Sigma |\times c$ pair of integers, which stores data needed by the inducing strategy discussed below. The values of *s*, *e*, *o* and *c* are constants that determine the amount of internal memory used in this phase.

The overall strategy used in Phase 2 (lines 10–20 of Algorithm 1) is the following. The first block of each $\mathsf {ESA} _i$ is loaded into the respective enhanced-array buffer $E_i$ (line 11). Then the heading element of each $E_i$ is inserted into a lexicographic minimum binary heap (line 12). Assume that the smallest suffix in the heap originates from $E_k$ (line 15). Then the suffix is moved to the output buffer (line 16), which is written to disk as it gets full (line 17–19), and the heap is filled with the next element in the buffer $E_k.$

Recall that during such comparisons the suffixes themselves are stored in external memory. Comparing suffixes in the heap may then require many random external memory accesses. To reduce external memory accesses, we propose an enhanced comparison method composed by three strategies: (a) prefix assembly, (b) $${ lcp} $$ comparison, and (c) suffix induction.

### Prefix assembly

Prefix assembly uses $\mathsf {PREFIX}$ arrays to retrieve portions of strings with no external memory accesses. These characters are those more likely to be needed to compare suffixes. Let *j* be the index of the smallest element in the enhanced-array buffer $E_i.$ The initial prefix of $T_i[\mathsf {SA} _i [j],n_i]$ may be loaded into $S_i$ by concatenating previous positions of $\mathsf {PREFIX} _i [k],$ for $k = 1,2,\dots ,j.$ As *j* changes, buffer $S_i$ is updated such that $S_i[1,h_{j}+p+1]=S_i[1,h_{j}]\cdot \mathsf {PREFIX} _i [j]\cdot \#,$ where $h_{j}=\min (\mathsf {LCP} _i [j],h_{j-1}+p),$ $h_0 = 0,$ and $\#$ is an end-of-buffer marker not in $\Sigma.$ Thus, if a string comparison does not involve more than $h_{j}+p$ symbols, an external memory access is not necessary. Otherwise $\#$ is reached and a portion of $T_i$ must be retrieved from the external memory. However, the part of $T_i$ that can be reconstructed from $\mathsf {PREFIX}$ is often long enough such that the first distinct characters can be accessed without I/O operations. In addition, the string buffer can easily and without great costs be adjusted to accommodate the relevant parts of $\mathsf {PREFIX},$ i.e. $h_{j}+p.$ Algorithm 2 illustrates prefix assembling applied to reconstruct the initial part of $T_i[\mathsf {SA} _i [k], n_i],$ for $k = 1,2,\ldots,j,$ into the string buffer $S_i[1,s].$ 
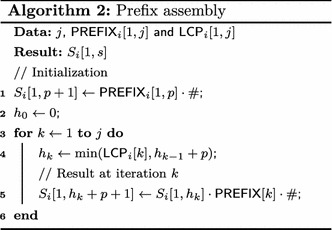


Column $${ suff} $$ in Table 3 illustrates the prefixes recovered by prefix assembly in bold. For example, for $$\mathsf {ESA} _1$$ shown in Table 4, when $$j=5$$ then $$h_5 = 0$$ and, since $$\mathsf {LCP} _1[5]=0,$$ $$S_1$$ stores $${ GA\$}.$$ When $$j=6$$ then $$h_6=\min (\mathsf {LCP} _1[6],h_5+p) = \min (2,0+3) = 2,$$ and $$S_1[3,3+3-1] = S_1[3,5]$$ receives $$\mathsf {PREFIX} _1[5] = { TAG}.$$ In this case, $$S_1 = S_1[1, 2]\cdot S_1[3, 5]\cdot \# = { GA\cdot TAG}\cdot \#= { GATAG\#}.$$

**Table 4:**
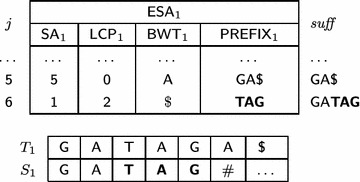
An example of a part of $\mathsf {ESA} _1$ illustrating the prefix assembly strategy

Symbols in bold highlight the substring of suffix $T_1[\mathsf {SA} [6], n_1]$ stored in $\mathsf {PREFIX} _1$

### $\mathsf {LCP}$ comparison

**Fig. 1:**
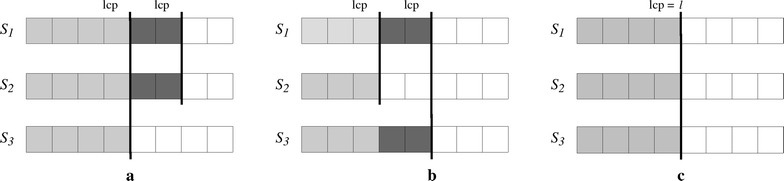
Illustration of Lemma 1. Illustration of the cases in the proof of Lemma 1: **a** case 1, **b** case 2 and **c** case 3

$${ lcp} $$ values can be used to speed up suffix comparisons [9, 43] and to avoid external memory accesses in heap insertions. The following lemma formalizes the idea. The proof is simple, based on the cases illustrated in Fig.  1, and will be omitted.

#### **Lemma 1**

*Let* $S_1,$ $S_2$ *and* $S_3$ *be strings, such that* $S_1<S_2$ *and* $S_1<S_3.$ *If* ${ lcp} (S_1,S_2)>{ lcp} (S_1,S_3)$ *then* $S_2<S_3$ *(case 1)*. *If* ${ lcp} (S_1,S_2)<{ lcp} (S_1,S_3)$ *then* $S_2>S_3$ *(case 2). Otherwise, if* ${ lcp} (S_1,S_2)={ lcp} (S_1,S_3)=\ell$ *then* ${ lcp} (S_2,S_3) \ge \ell$ *(case 3)*.

Let *X*, *Y* and *Z* be nodes in the binary heap storing $E_a[i],$ $E_b[j]$ and $E_c[k],$ respectively. Let *X*, *Y* and *Z* be also the suffixes stored by such heap nodes. Suppose that node *X* is the parent of *Y* and *Z*. Because $X<Y$ and $X<Z$ it follows that $T_a[\mathsf {SA} _a[i],n_a]<T_b[\mathsf {SA} _b[j],n_b]$ and $T_a[\mathsf {SA} _a[i],n_a]<T_c[\mathsf {SA} _c[k],n_c].$ Assume that the heap also stores ${ lcp}$ values between a node and its children and between a node and its sibling.

As *X* is removed from the heap, $E_a[i]$ is moved to the output buffer and *X* is replaced by another node *W* storing $E_a[i+1].$ The order of *W* with respect to its children *Y* and *Z* can be determined without character comparisons when case 1 or case 2 of Lemma 1 applies, and if case 3 applies then the character comparison can be started from symbol $\ell = { lcp} (X,W),$ recalling that ${ lcp} (X,W)$ is stored in $E_a[i+1].$ In the same way the order between *Y* and *Z* can be determined using Lemma 1. Algorithm 3 illustrates this procedure to compare the nodes *W*, *Y* and *Z* in the heap.
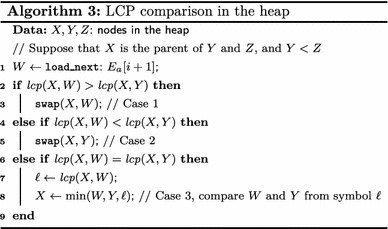


${ lcp}$ values between nodes in the heap are updated as nodes are compared and swapped. Suppose that node *W* is swapped with *Y* (meaning $Y<W$ and $Y<Z$). The ${ lcp}$ of *W* with respect to its new children are also determined using Lemma 1, taking the minimum ${ lcp}$ between two suffixes (in cases 1 and 2) or through direct character comparisons (case 3). Hence, by using ${ lcp}$ values many direct comparisons of strings that are in external memory are avoided.

For instance, consider merging $\mathsf {ESA} _1$ and $\mathsf {ESA} _2$ in Table 1. First, comparing the elements $\mathsf {ESA} _1[4]$ and $\mathsf {ESA} _2[3]$ we conclude that ${ suff} _2(3) = { AGA\$}$ is less than ${ suff} _1(4) = { ATAGA\$}.$ The next comparison involves $\mathsf {ESA} _1[4]$ and $\mathsf {ESA} _2[4].$ As already stated, without comparing any symbols we see that ${ lcp} ({ suff} _2(3),{ suff} _2(4)) > { lcp} ({ suff} _2(3),{ suff} _1(4))$ and that ${ suff} _2(4) = { AGAGA\$}$ is less than ${ suff} _1(4) = { ATAGA\$}.$

### Suffix induction

The induced sorting principle corresponds to deduce the order of unsorted suffixes from already sorted suffixes. This strategy is used by many suffix array construction algorithms [49]. We apply an induced sorting approach that relies on the following lemma. Let a suffix starting with a symbol $\alpha$ be denoted $\alpha$-suffix and let ${ suff}_{\mathcal {T}}$ be the set of all suffixes of strings in $\mathcal {T}$.

#### **Lemma 2**

*If* $T_i[j,n_i]$ *is the smallest suffix in* ${ suff}_{\mathcal {T}}$ *then* $T_i[j-1,n_i] = \alpha \cdot T_i[j, n_i]$ *is the smallest* $\alpha$ *-suffix* *in* ${ suff}_{\mathcal {T}} \setminus \{T_i[j,n_i]\}.$

#### *Proof*

Suppose that there is a $\alpha$ *-suffix* $T_{\ell }[k,n_{\ell }]$ in ${ suff}_{\mathcal {T}}$ that precedes $T_i[j-1,n_i].$ Then $T_{\ell }[k+1,n_{\ell }]$ must be smaller than $T_i[j,n_i],$ a contradiction.

Lemma 2 can be used for sorting the suffixes of a string *T* of length *n* as follows. Let an $\alpha$-bucket be a block of a partition of $\mathsf {SA}$ that contains only $\alpha$-suffixes. ${ suff}_{\mathcal {T}}$ is initialized with every suffix of *T* and an empty bucket for each symbol in $\Sigma$ is created. While ${ suff}_{\mathcal {T}}$ is not empty, the smallest suffix $T[j,n]=\alpha \cdot T[j+1,n]$ in ${ suff}_{\mathcal {T}}$ is moved to the leftmost available position in the $\alpha$-bucket and, if $\alpha <\beta$ then $T[j-1,n]=\beta \cdot T[j,n]$ is added to the leftmost available position in the $\beta$-bucket (it is induced). The induced suffix $T[j-1,n]$ cannot be removed from ${ suff}_{\mathcal {T}}$ yet because it may induce $T[j-2,n_i]$ as well. When a suffix that is already in a bucket is also the smallest in ${ suff}_{\mathcal {T}},$ the suffix itself and those that succeed it in the bucket are used to induce another suffix and are removed from ${ suff}_{\mathcal {T}}$ at once. Note that if $\alpha >\beta$ then the suffix $T[j-1,n_i]$ was already sorted and if $\alpha =\beta$ then reading induced suffixes from the $\beta$-bucket can cause the induction of already induced suffixes. So no induction is done when $\alpha \ge \beta.$

This approach is not efficient to sort the suffixes of a single string *T*, since it is often necessary to find a smallest suffix. But in merging previously sorted suffixes the smallest one can be determined efficiently using the heap. Suppose that $E_i[k]$ is at the root of the heap. Then $T_i[j,n_i]$ is the smallest suffix in ${ suff}_{\mathcal {T}}$ and $T_i[j-1,n_i]$ can be induced if $T_i[j]<T_i[j-1].$ This later test may be performed using $\mathsf {BWT} _i$ and, as a consequence, to determine whether $T_i[j-1,n_i]$ can be induced or not.

Induced suffixes are added to the induced buffer *I*, partitioned into buckets $I_{\alpha },$ one for each $\alpha \in \Sigma.$ When an $\alpha$-suffix from string $T_i$ is induced, the value *i* is inserted into the first available position of $I_{\alpha },$ which is written to an external memory file $F_{\alpha }$ as it gets full. When the smallest $\alpha$-suffix is at the root of the heap, $F_{\alpha }$ is read sequentially to retrieve string indexes. Each string index *i* indicates that the smallest suffix in $E_i$ may be written to the output directly, since such suffix has been induced, bypassing operations in the heap and saving many comparisons. When every index in $F_{\alpha }$ has been processed the heap must be reconstructed. Algorithm 4 illustrates Phase 2 (see Algorithm 1) augmented for suffix induction. Whenever the first suffix starting with $\alpha = T_a[b]$ is returned from the heap, $\mathsf {eGSA}$ induces the output buffer the suffixes in $F_{\alpha }.$ 
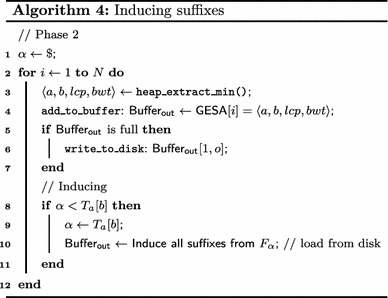


$${ lcp} $$ values for induced suffixes must also be induced, since induced suffixes are not compared in the heap. Suppose that $$T_a[i,n_a]$$ induces an $$\alpha $$-suffix and suppose that $$T_b[j,n_b]$$ induces the next $$\alpha $$-suffix. Then $$\mathsf {LCP} (T_a[i-1,n_a],T_b[j-1,n_b]) = \mathsf {LCP} (T_a[i,n_a],T_b[j,n_b])+1.$$ But since $$T_a[i,n_a]$$ and $$T_b[j,n_b]$$ may not be consecutive in $$\mathsf {GSA}, $$ $$\mathsf {LCP} (T_a[i,n_a],T_b[j,n_b])$$ may not be obtained directly. Such value may be obtained from the range minimum query on the $$\mathsf {LCP},$$ defined as $${ rmq} (x,y)=\min _{x \le k \le y} \{\mathsf {LCP} [k]\}.$$ It is easy to see that as $$T_a[i,n_a]$$ and $$T_b[j,n_b]$$ are already sorted and $$\mathsf {LCP} (T_a[j,n_a],T_b[j,n_b]) = { rmq} ({ pos} (T_a[j,n_a]+1),{ pos} (T_b[j,n_b]))$$ the $${ rmq}$$ value may be computed as $$\mathsf {LCP} $$ values are moved to the output buffer.

Therefore, when a suffix $$T_i[j,n_i]$$ is induced in the second phase, its corresponding $$\mathsf {LCP} $$ is also induced. As induced suffixes may also induce further suffixes, the corresponding $$\mathsf {LCP} $$ must be stored in the induced buffer $$I_{\alpha }$$ and in the respective file as well. As induced suffixes are recovered from external memory, $$\mathsf {LCP} $$ values are recovered to update the $${ rmq} $$ computation.

For instance, suppose that $$T_1[6,n_1] = A\$ $$ is the smallest suffix in the heap during the merge of $$\mathsf {ESA} _1$$ and $$\mathsf {ESA} _2$$ in Table 1. Because $$\mathsf {ESA} _1[2].\mathsf {BWT} = G>A,$$ $$T_1[6-1=5,n_1]={ GA\$}$$ is induced as the smallest *G*-suffix in $${ suff}_{\mathcal {T}}.$$ Then the pair (1, 0) is written to the buffer $$I_G$$ to indicate that a suffix from string 1 was induced with $${ lcp} = 0.$$ The $${ lcp}$$ value in $$\mathsf {GESA}$$ between $$T_1[5,n_1]$$ and the next induced *G*-suffix ($$T_2[5, n_2]$$) is computed by the minimum $${ lcp} $$ value from the suffixes passing through the heap until $$T_2[5, n_2]$$ is induced. This happens when $$T_2[6,n_2]$$ is the smallest element in the heap and $$T_2[5, n_2]$$ is induced together with the $${ lcp} (T_1[6, n_1] ,T_2[6, n_2])+1 = 2,$$ obtained by the current minimum $${ lcp} $$ value. When $$T_1[5,n_1]$$ is the smallest suffix in the heap, $$F_{G}$$ is read sequentially and the induced *G*-suffixes are recovered together with their $${ lcp}$$ values.

Using prefix assembly together with induction requires additional care. Since induced suffixes are not compared in the heap, they do not participate in the prefix assembly. Thus during the evaluation of $\mathsf {PREFIX}$ in Phase 1, $h_{j}$ must be equal to 0 for every last $\alpha$-suffix that will be induced, then the prefix of the first non-induced $\alpha$-suffix will start at its initial position. To this end, we set 0 as the $\mathsf {LCP} [pos(T_i[j,n_i])]$ of every suffix $T_i[j,n_i]$ that will be induced, i.e. when $T_i[j] > T_i[j+1].$ Recall that all such ${ lcp}$ values will be also induced.

For instance, Table 5 illustrates the construction of $\mathsf {ESA} _1$ in the first phase of $\mathsf {eGSA},$ for $j=2.$ When $j = 2,$ $\mathsf {SA} [j=2] = 6$ and $T_1[6]>T_1[6+1],$ then the suffix $T_1[6,n_1]$ will be induced and $\mathsf {LCP} _1[2+1=3]$ receives 0. Next, $j = 3,$ $\mathsf {SA} [j=3] = 4$ and $T_1[4]<T_1[4+1],$ the suffix $T_1[4,n_1]$ will not be induced. It means that, in the second phase, $T_1[6,n_1]$ will be induced and bypassed in the heap, thus the prefix assembling of suffix $T_1[4,n_1]$ must start from scratch in $S_1$. From this point, prefix assembly continues normally.

**Table 5:**
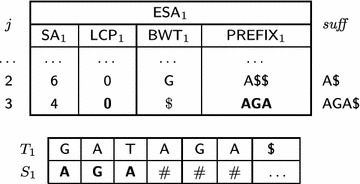
Prefix assembly and inducing suffixes

Symbols in bold illustrate the substring of suffix $T_1[SA[3], n_1]$ stored in $\mathsf {PREFIX}$

### Theoretical costs

Phase 1 of $\mathsf {eGSA}$ is dominated by the algorithms used to construct $\mathsf {SA}$ and $\mathsf {LCP}.$ The other columns of the generalized suffix array are evaluated when the output is written to disk, using constant time and memory per item. The construction of $\mathsf {SA}$ and $\mathsf {LCP}$ may be done in linear time and space [29, 46]. Thus, for *m* input strings with total length *N* and $T_{\ell }$ the longest string, Phase 1 is $O(m|T_{\ell }|)$ time plus *O*(*N*) I/O operations using $O(|T_{\ell }|)$ memory.

In Phase 2, the number of node swaps in the heap is bounded by $$N\log m.$$ Each node swap requires comparing a number of characters that is at most the maximum value of $${ lcp} $$ for $$\mathcal {T}$$ ($${ maxlcp} $$). The time cost of this phase is then $$O((N\log m){ maxlcp}).$$ I/O operations in Phase 2 include loading portions of suffix arrays and of strings from disk, and writing output buffers to disk. Suffix arrays are loaded in blocks to the enhanced-array buffers. In the worst case each comparison in the heap will trigger a character comparison, and the string buffers will be loaded when exhausted. Provided that the string buffer is at least as large as $${ maxlcp} ,$$ each suffix will cause at most one I/O operation and the worst case for the number of string buffer load operations is *O*(*N*). The number of I/O operations on enhanced-array and output buffers is limited by *N* divided by the respective buffer sizes. Then the number of I/O operations in Phase 2 is bounded by *O*(*N*). The memory usage in Phase 2 is bounded by the sum of buffer sizes, which can be tailored as necessary.

Such bounds for I/O operations are prohibitive, but it is much lower in practice due to the optimizing strategies, as shown in the next sections. An easy to devise limitation of $\mathsf {eGSA}$ is the case of datasets whose strings are large and highly repetitive, for instance, a dataset composed by human genomes of different individuals. For these datasets the practical performance will approach the theoretical bound. Another limitation is when ${ maxlcp}$ is larger than the string buffer size, when the number of I/O operations is as bad as $O(N \log m (|T_{\ell }|/s)),$ where *s* is the string buffer size.

## Performance evaluation

We used four real datasets of different domains, including DNA and protein sequences, and natural language texts as described in Table 6. The table includes the total size of each dataset in GB, the number of strings, the average string length, and the average and maximum ${ lcp}$ values, which provide an approximation of suffix sorting difficulty [16].

**Table 6:** Datasets used in the experiments

Dataset	Size (GB)	Number of strings	Total length	Avg. length	Max. lcp	Avg. lcp
dna	9.85	153	10,580,043,054	69,150,608	2,282,187	1122
protein	18.68	62,148,086	20,056,474,339	323	31,815	88
gutenberg	22.32	407,864,056	23,962,356,903	59	11,946	18
enwiki	24.50	351,363,467	25,648,226,940	75	111,273	33

**dna:**a collection of large DNA chromosomes from organisms (*Homo sapiens*, *Oryzias latipes*, *Danio rerio*, *Bos taurus*, *Mus musculus* and *Gallus gallus*) of Ensembl dataset (ftp://ftp.ensembl.org/pub/release-84/fasta/). We removed any occurrences of the character N (unknown) from the strings

**protein:** the collection of protein sequences from Uniprot/TrEMBL, release 2016_5 (http://www.ebi.ac.uk/uniprot/download-center/)

**gutenberg:** a collection of documents from Gutenberg Project, release 2012_09 (http://algo2.iti.kit.edu/bingmann/esais-corpus/). We processed each line of the input as a single string

**enwiki:** a collection of pages from a snapshot of the English language edition of Wikipedia release 2016_05 (https://dumps.wikimedia.org/enwiki/20160501/). We processed each line of the input as a single string

The experiments were conducted on Debian GNU/Linux 6.0.3/64 bits operating system using an Intel(R) Xeon(R) CPU E3-1230 V2 @ 3.30 GHz processor 8 MB cache, with 32 GB of internal memory and a 2.0 TB SATA hard disk with 7200 RPM and 64 MB cache (Seagate Desktop HDD ST2000DM001). Our algorithm was implemented in ANSI/C and compiled by GNU GCC version 4.6.3, with optimizing option -O3. The source code is freely available at https://github.com/felipelouza/egsa/.

In Phase 1 we partitioned the collection of strings $\mathcal {T}$ into *k* groups, such that when the strings in each group are concatenated the resulting string $T^{cat}$ may be given to internal memory $\mathsf {SA}$ and $\mathsf {LCP}$ construction algorithms. After concatenating the strings in a group a new terminator symbol $\#$ that is smaller than $\$$ is added to the end of $T^{cat}.$ For the first phase we used gSACA-K [36] combined with $\Phi$-algorithm [29]. gSACA-K guarantees that the order of equal suffixes from different strings in a group will be defined by the rank of their strings in $\mathcal {T}$. Given the $\mathsf {SA}$ of $T^{cat},$ we compute $\mathsf {GSA}$ for the string group using an additional integer array *DA* of size $|T^{cat}|$ that stores in *DA*[*i*] the string to which suffix $T^{cat}[i, |T^{cat}|]$ belongs in $\mathcal {T}.$ *DA* can be computed easily by scanning $T^{cat}.$ Then, each value $\mathsf {SA} [i]$ is mapped to $\mathsf {GSA} [i].{ str}$ and $\mathsf {GSA} [i].{ suff} ,$ and the $\mathsf {GSA}$ for the string group is written to external memory. $\mathsf {ESA} [i],$ that will be used in Phase 2, will be composed by $\langle \mathsf {GSA} [i],\mathsf {LCP} [i],\mathsf {BWT} [i],\mathsf {PREFIX} [i]\rangle.$ The $\Phi$-algorithm was adapted to stop the comparison in $T^{cat}$ when it reaches $\$$ symbols, thus correctly evaluating the $\mathsf {LCP}$ between suffixes in the same group. Together, these algorithms use $13 \times |T^{cat}|$ bytes. In this experiments, when $T^{cat}$ is composed by only one string $T_{\ell }$ and $13 \times |T^{cat}|$ is larger than the available internal memory, the algorithm truncates $T_{\ell },$ such that $13 \times |T^{cat}|$ fits in memory. The sizes reported in Table 6 refer to the datasets after truncations, that happened only with dna.

In Phase 2 we used $p=10$ for the prefix array size, which provided a good tradeoff between time and disk usage space, as shown in "eGSA internals" section. Each buffer $S_i$ were set to use 20 KB of internal memory, whereas all buffers *B*, $\mathsf {Buffer_{out}}$ and *I* were set to use 1 GB, 64 MB and 16 MB, respectively, in total. We remark that $\mathsf {eGSA}$ uses 1 byte to store each character in memory. The output produced by $\mathsf {eGSA}$ was validated using a trivial checking algorithm.

In "Relative performance" section we investigate the behavior of $\mathsf {eGSA}$ with respect to $\mathsf {eSAIS}$  [10] and $\mathsf {SAscan}$  [27]. In "eGSA internals" section we evaluate $\mathsf {eGSA}$ in detail, showing the influence of each phase and of the improving strategies used in Phase 2 on the total running time. In "Limitations" section we investigate limitations of our algorithm related to the effect of disk cache managed by the operating system when the internal memory (RAM) size is restricted at boot time.

### Relative performance

To assess the performance of $\mathsf {eGSA}$ we compared it to $\mathsf {eSAIS}$  [11], which is the fastest algorithm to date to compute both suffix and $\mathsf {LCP}$ arrays in external memory. We also compared $\mathsf {eGSA}$ to $\mathsf {SAscan}$  [28], which computes only the suffix array with small peak disk usage. We configured the algorithms to use the same disk for input and output. We are aware of the existence of the algorithms by Bauer et al.  [5, 6] and by Cox et al.  [13] that aim at indexing collections of small strings in external memory. However, we did not consider comparing them with $\mathsf {eGSA}$ because they were designed to solve a different problem, namely building the $\mathsf {BWT}$ and the $\mathsf {LCP}$ array with small memory footprint. Moreover, a comparison in the article [13] have shown that $\mathsf {eGSA}$ is faster and uses more space in external memory.

Although $\mathsf {eSAIS}$ and $\mathsf {SAscan}$ are aimed at indexing only one string, we can concatenate all strings and use $\mathsf {eSAIS}$ or $\mathsf {SAscan}$ to construct the generalized suffix arrays. All strings in $\mathcal {T}$ were concatenated and a final terminator $\#$ was added, such that $\# < \$$. This concatenation strategy will not guarantee that equal suffixes will be sorted by string rank and the values in $\mathsf {LCP}$ may be larger than the actual ${ lcp}$ of consecutive suffixes in $\mathsf {GESA},$ but will not impose the growth of the alphabet size and still allows $\mathsf {eSAIS}$ and $\mathsf {SAscan}$ to use 1 byte per input character.

We remark that the results presented in this section depends on the RAM size available in the experiments, that is, 32 GB. As we show in "Limitations" section, the performance and efficiency of $\mathsf {eGSA}$ degrades as the total RAM size is reduced.

#### Running time and efficiency

**Fig. 2:**
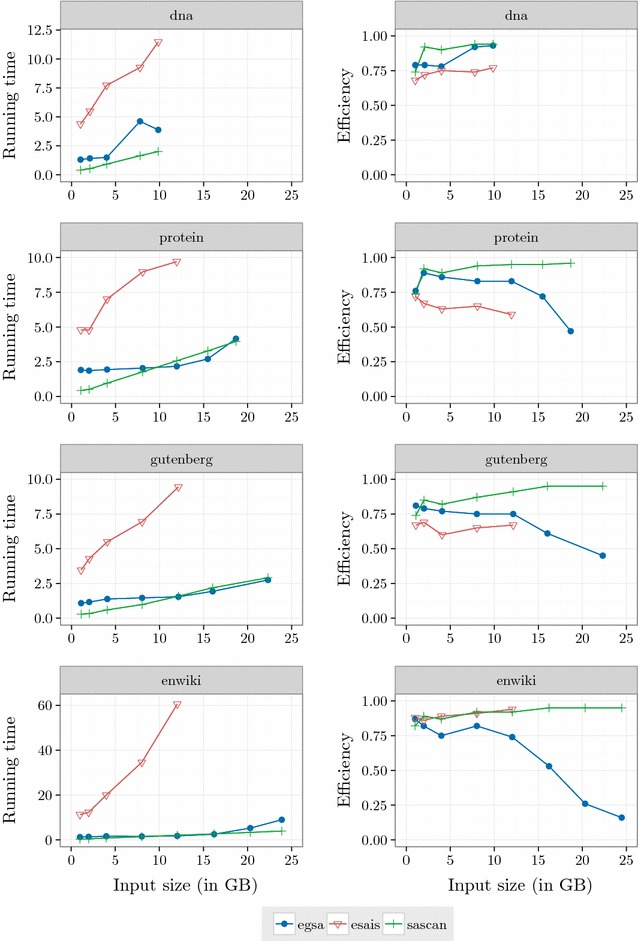
Running time. Running time in microseconds per input byte and the efficiency of $\mathsf {eGSA},$ $\mathsf {eSAIS}$ and $\mathsf {SAscan}.$ Efficiency is the proportion of time for which the CPU is busy, not waiting for I/O. The running time of $\mathsf {eGSA}$ is consistently smaller than that of $\mathsf {eSAIS}$ and comparable to $\mathsf {SAscan}.$ Recall that $\mathsf {SAscan}$ computes only the $\mathsf {SA}$

Figure 2 shows the running time in microseconds per input byte and the efficiency of $\mathsf {eGSA},$ $\mathsf {eSAIS}$ and $\mathsf {SAscan}.$ Efficiency is the proportion of time for which the CPU is busy, not waiting for I/O. Except for dna, $\mathsf {eSAIS}$ was interrupted for datasets with more than 12 GB due to the large amount of time to process these instances. For example, $\mathsf {eSAIS}$ took 9 days to run on enwiki with 12 GB. The experiments took about 70 days of computing to finish.

The amount of internal memory used by the algorithms is an input parameter. We configured them to use 2 GB. Although the comparison is not totally fair because $\mathsf {eSAIS}$ and $\mathsf {SAscan}$ were not designed for multiple strings, $\mathsf {eGSA}$ have outperformed $\mathsf {eSAIS}$ and presented a competitive performance compared to $\mathsf {SAscan},$ which computes only the $\mathsf {SA}.$ Moreover, $\mathsf {eGSA}$ can also construct the generalized $\mathsf {BWT}$ of the collection $\mathcal {T}$ with no additional cost except by the output time.

The long running times of $\mathsf {eSAIS}$ prevented the analysis of its efficiency trend. In the extreme case, enwiki with 12 GB, the running time of $\mathsf {eSAIS}$ is almost 35 times larger than the time spent by $\mathsf {eGSA}.$ The running times of $\mathsf {eGSA}$ and $\mathsf {SAscan}$ are very close, with larger differences only for the dna dataset. $\mathsf {SAscan}$ presents the best efficiency, which is mostly unaffected by the size of the dataset. The efficiency of $\mathsf {eGSA}$ is comparable to $\mathsf {SAscan}$ for small datasets and better than the efficiency of $\mathsf {eSAIS}.$ The efficiency of $\mathsf {eGSA}$ drops with the size of the dataset. For larger datasets it becomes apparent that the efficiency of $\mathsf {eGSA}$ is strongly affected by the effect of the disk cache managed by the operating system, since the size of the available internal memory decreases as the dataset increases (we evaluate this issue in "Limitations" section).

#### I/O volume and peak disk usage

**Fig. 3:**
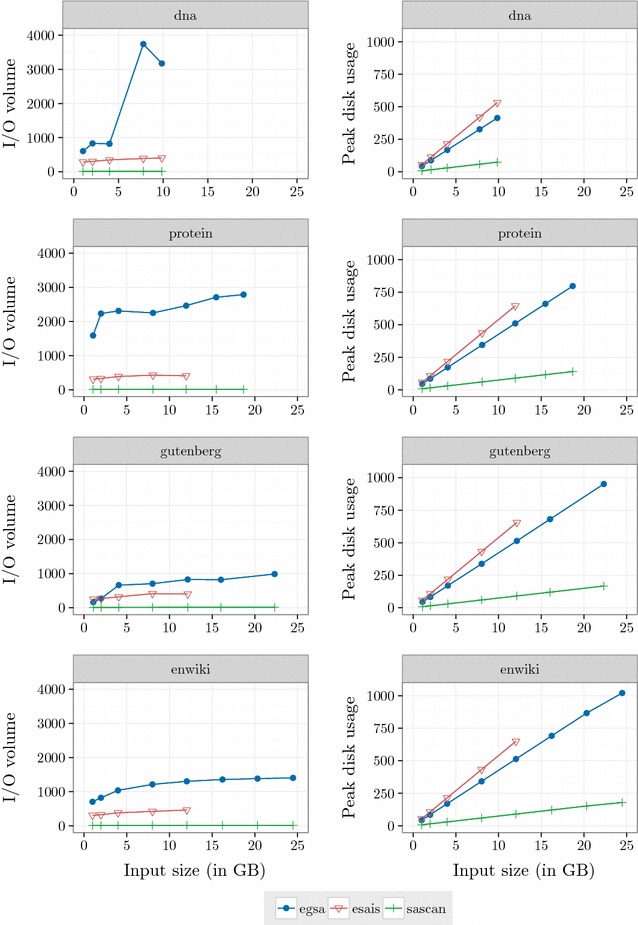
I/O volume. I/O volume (in bytes per input byte) and the peak disk usage (in GB) of $\mathsf {eGSA},$ $\mathsf {eSAIS}$ and $\mathsf {SAscan}$

The I/O volume (in bytes per input byte) and the peak disk usage (in GB) of each algorithm are reported in Fig.  3. $\mathsf {eGSA}$ makes a larger volume of I/O transfer. In the extreme case, protein with 12 GB, $\mathsf {eGSA}$ transfer more than 6 times data than $\mathsf {eSAIS}$ and $\mathsf {eGSA}$ transfers 150 times more data than $\mathsf {SAscan}.$ $\mathsf {eGSA}$ uses 39*n* bytes (8*n* bytes for $\mathsf {GSA}$, 4*n* bytes for $\mathsf {LCP},$ and 27*n* bytes for auxiliary structures) plus by the size of the temporary files used to store induced suffixes. As can be seen in Fig.  5, the average number of induced suffixes is about $43\%,$ and is almost constant for all dataset sizes. $\mathsf {eSAIS}$ uses 54*n* bytes to compute $\mathsf {SA}$ and $\mathsf {LCP}$ arrays, whereas $\mathsf {SAscan}$ uses 7.5*n* bytes to compute $\mathsf {SA}.$ Overall, the peak disk usage is much smaller for $\mathsf {SAscan}$.

Although $\mathsf {eSAIS}$ and $\mathsf {SAscan}$ do not take care of the peculiarities of a generalized suffix array, $\mathsf {eGSA}$ still shows faster or comparable running times. Therefore, $\mathsf {eGSA}$ is a good alternative for the construction of the generalized enhanced suffix array in external memory.

### $\mathsf {eGSA}$ internals

We have evaluated the behavior of $\mathsf {eGSA}$ in terms of the performance of each phase and the effect of each heap strategy used in Phase 2.

**Fig. 4:**
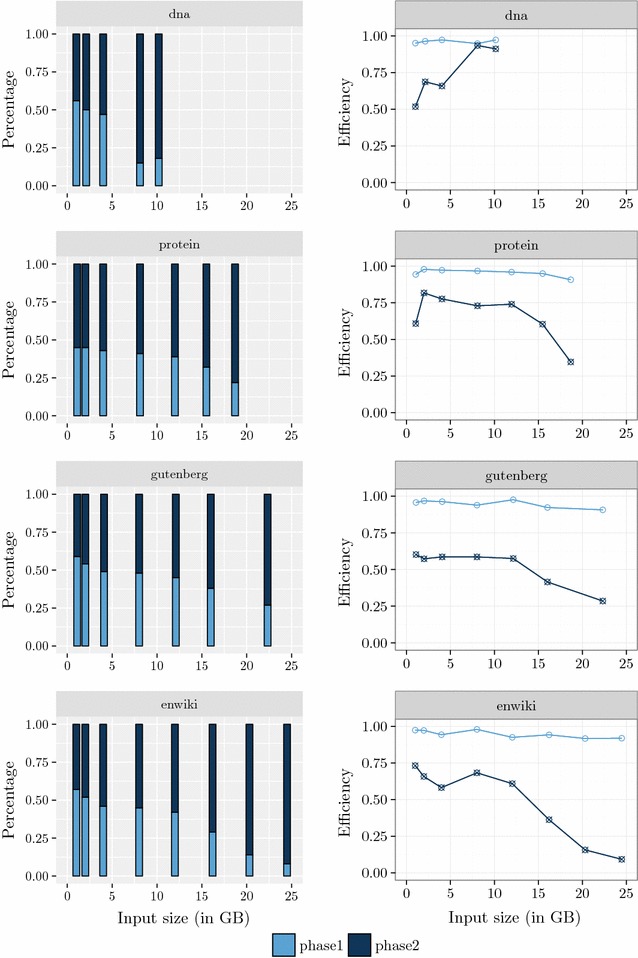
Running time of each phase. Percentage of the running time of each $\mathsf {eGSA}$ phase and its efficiency

Figure 4 shows the percentage of time spent by each phase of $\mathsf {eGSA}$ and its efficiency. We can see that the percentage of the time spent by Phase 2 increases as the dataset increases and dominates the time of $\mathsf {eGSA}$. We can see that the efficiency of Phase 1 is almost constant and the efficiency of Phase 2 is better for small alphabets (dna).

**Fig. 5:**
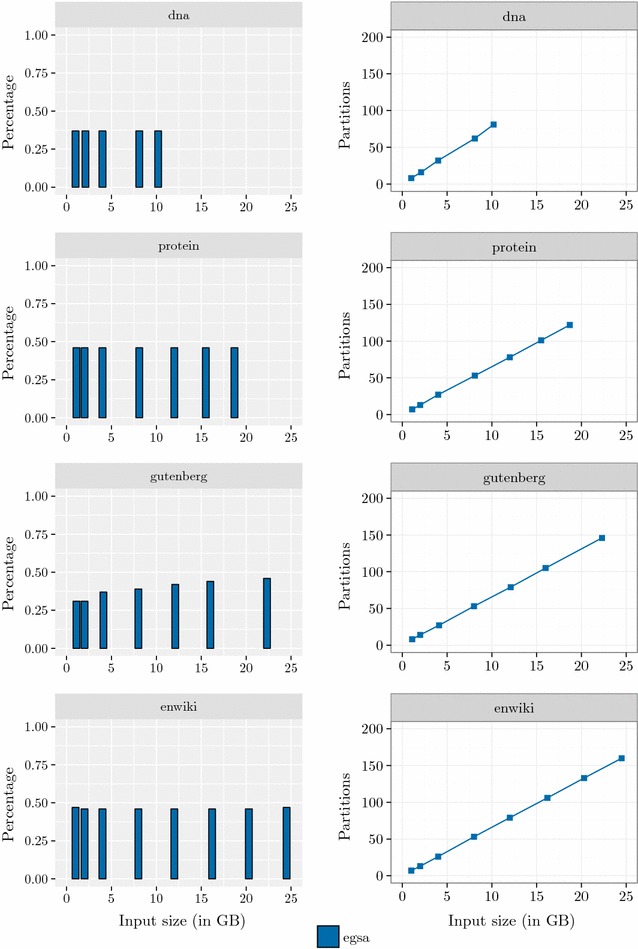
Induced suffixes and partitions. Percentage of induced suffixes and the number of partitions created by $\mathsf {eGSA}$

Figure 5 shows the percentage of induced suffixes and the number of partitions created by $\mathsf {eGSA}$ in the preprocessing step. In the average, $42\%$ of the suffixes were induced. This indicates that the algorithm is avoiding many string comparisons. The number of partitions grows linearly with the dataset size, and the figure shows Phase 1 using less than 2 GB.

#### Prefix array size

We have analyzed the effect of the value of the parameter *p* on the running time. We used the first 8 GB of each dataset for these experiments. Recall that *p* is the number of symbols in each position of $\mathsf {PREFIX}$ arrays and has a major impact on external memory usage and access. As the value of *p* grows the external memory access decreases but the peak disk space usage increases. We evaluated some values for *p* with fixed memory usage, that is, increasing *p* implied an reduction of the number of elements in the partition buffers $B_i$, guaranteeing that all versions use the same amount of internal memory. Table 7 shows the effect of *p* on the total running time and the efficiency of $\mathsf {eGSA},$ for *p* varying between 0 and 25. The value $p=10$ resulted in a good tradeoff between the peak disk space used by the algorithm and the running time.

**Table 7:** Time spent by $\mathsf {eGSA}$ according to the prefix array size

Dataset	$p=0$	$p=5$	$p=10$	$p=15$	$p=20$
Time in $\upmu$s/byte					
dna.8GB	5.68	4.97	4.91	4.48	5.03
protein.8GB	2.58	2.00	2.04	2.00	2.12
gutenberg.8GB	2.33	1.66	1.46	1.48	1.33
enwiki.8GB	2.25	1.87	1.54	1.64	1.48
Efficiency					
dna.8GB	0.97	0.81	0.93	0.93	0.83
protein.8GB	0.92	0.87	0.83	0.82	0.76
gutenberg.8GB	0.92	0.78	0.75	0.69	0.74
enwiki.8GB	0.92	0.80	0.82	0.70	0.74

The experiment with $p=10$ is the same presented in Figs.  2, 3, 4 and 5 and $p=0$ means that the prefix assembly strategy was not used by the algorithm

#### Effect of optimizations

In order to evaluate the effect of strategies that help to avoid character comparisons in $\mathsf {eGSA},$ namely (a) prefix assembly, (b) $\mathsf {LCP}$ comparison and (c) suffix induction, every possible combination of them was tested. Again, we used the first 8 GB of each dataset. The running time and the efficiency for each dataset is shown in Table  8.

We can see that the complete version of $\mathsf {eGSA}$ (column $\{a, b, c\}$) was the best in the majority of the cases. The dataset gutenberg.8GB was faster using $\{a, b\}$ with a difference of about 10%. Comparing with the $\emptyset$ version, optimization strategies reduced the time by a factor of 1.9−2.22. Note that all strategies individually improved performance with respect to the $\emptyset$ version. Thus, we may conclude that the use of heap strategies heavily improves the performance of $\mathsf {eGSA}.$ As a final remark, we note that, except for dna.8GB, the $\emptyset$ version reduced the time by a factor of up to 6.10 compared with $\mathsf {eSAIS}$ (Fig. 2).

**Table 8:** Effect of each heap strategies on time

Dataset	$\emptyset$	{a}	{b}	{c}	{a, b}	{a, c}	{b, c}	{a, b, c}
Time in $\upmu$s/byte								
dna.8GB	10.08	8.13	8.52	6.79	6.43	5.60	5.68	4.91
protein.8GB	3.88	2.74	3.49	2.76	2.26	2.15	2.58	2.04
gutenberg.8GB	3.18	1.48	2.96	2.47	1.35	1.55	2.33	1.46
enwiki.8GB	3.28	1.87	3.04	2.42	1.73	1.82	2.25	1.54
Efficiency								
dna.8GB	0.98	0.97	0.98	0.98	0.95	0.95	0.97	0.93
protein.8GB	0.95	0.88	0.96	0.94	0.89	0.87	0.92	0.83
gutenberg.8GB	0.95	0.83	0.95	0.93	0.77	0.77	0.92	0.75
enwiki.8GB	0.96	0.86	0.95	0.91	0.78	0.76	0.92	0.82

All possible combinations of (a) prefix assembly, (b) $\mathsf {LCP}$ comparison and (c) suffix induction are plotted for the datasets. $\emptyset$ is the case when none of them is used, and $\{b, c\}$ and $\{a, b, c\}$ are the same presented in columns $p=0$ and $p=10$ of Table 7

### Limitations

We have investigated the effect of the disk caching in the performance of $\mathsf {eGSA},$ $\mathsf {eSAIS}$ and $\mathsf {SAscan}.$ We restricted both the internal memory available to our algorithm (2 GB) as well as the total system memory at boot time (8−24 GB).

Table 9 shows the running time and the efficiency of each algorithm set to use 2 GB of internal memory to process the first 8 GB of the dataset dna in a machine whose total RAM was restricted to 24, 16, 12, 10 and 8 GB at boot time. The values in the last column (32 GB) are the same presented in "Relative performance" and "eGSA internals" sections. We also tested the datasets protein.8GB, gutenberg.8GB and enwiki.8GB and we obtained very close results, which were omitted.

The running time and efficiency of each algorithm degrade as the total RAM size reduces. This happens as an effect of the reduction of free memory available for disk caching managed by the operating system, which reduces the number of disk accesses. Comparing to the original setting (32 GB), the running time of $\mathsf {eGSA}$ was about 25 times larger with the RAM size restricted to 8 GB, whereas for $\mathsf {eSAIS}$ and $\mathsf {SAscan}$ their running times were about 1.3 larger.

For $\mathsf {eGSA},$ disk caching reduces disk accesses to the input strings as suffixes are moved along the heap, which displays a “random” pattern. This is where the worst case complexity stated in "Theoretical costs" section shows its claws. On the other hand, $\mathsf {eGSA}$ takes advantage of the disk cache system, which might be a favorable aspect in practical setups. Recall that the total size of the output data structure is 12 times the dataset size, which is 96 GB for the dataset dna.8GB.

The experiments show that $\mathsf {eGSA}$ depends on the availability of a large amount of free RAM to be efficient, which can be seen as a feature of a semi-external algorithm [8]. However, $\mathsf {eGSA}$ works purely in external memory. We believe that the optimizing strategies applied on the heap are interesting *per se*, and, as the disk access pattern is not actually random, may be there is still room for improving the overall strategy based on a heap, what could improve the performance of $\mathsf {eGSA}$ with less support of disk caching.

**Table 9:** Time spent by $\mathsf {eGSA},$ $\mathsf {eSAIS}$ and $\mathsf {SAscan}$ to process dna.8GB according to the total RAM size

Algorithm	8 GB	10 GB	12 GB	16 GB	24 GB	32 GB
Time in $\upmu$s/byte						
$\mathsf {eGSA}$	112.89	34.36	10.14	5.65	4.63	4.60
$\mathsf {eSAIS}$	12.17	11.16	11.55	10.62	11.39	9.36
$\mathsf {SAscan}$	2.10	1.71	1.83	1.70	1.59	1.65
Efficiency						
$\mathsf {eGSA}$	0.05	0.33	0.43	0.76	0.92	0.92
$\mathsf {eSAIS}$	0.66	0.65	0.63	0.68	0.64	0.74
$\mathsf {SAscan}$	0.86	0.90	0.88	0.94	0.94	0.94

## Conclusions

In this article we proposed $\mathsf {eGSA},$ which is the first external memory algorithm to construct generalized suffix arrays enhanced with the longest common prefix array ($\mathsf {LCP}$) and the Burrows–Wheeler transform ($\mathsf {BWT}$) for a collection of strings. The proposed algorithm was validated through performance tests using real datasets from different domains, in various combinations. Compared to $\mathsf {eSAIS}$ and $\mathsf {SAscan},$ $\mathsf {eGSA}$ showed a competitive performance. Moreover, our algorithm can also constructs the generalized $\mathsf {BWT}$ of a collection of strings with no additional cost except by the output time.

Another advantage of $\mathsf {eGSA}$ is that it may be employed to build generalized enhanced suffix arrays from arrays that have already been computed individually for strings in a dataset. Moreover, $\mathsf {eGSA}$ may be used to construct the core data structures used by LOF-SA [50] and ROSA search algorithms [21], or to build generalized suffix trees in external memory [4]. Furthermore, it may be applied to solve the longest common substring problem [1, 3] and to construct the Longest Previous Factor array, which is used in text compression and for detecting motifs and repeats [15].

## References

[1] Arnold M, Ohlebusch E (2011). Linear time algorithms for generalizations of the longest common substring problem. Algorithmica.

[2] Abouelhoda MI, Kurtz S, Ohlebusch E (2004). Replacing suffix trees with enhanced suffix arrays. J Discret Algorithms.

[3] Babenko MA, Starikovskaya T. Computing longest common substrings via suffix arrays. In: Proceedings of computer science in Russia symposium. 2008. p. 64–75.

[4] Barsky M, Stege U, Thomo A (2011). Suffix trees for inputs larger than main memory. Inform Syst J.

[5] Bauer MJ, Cox AJ, Rosone G (2013). Lightweight algorithms for constructing and inverting the BWT of string collections. Theor Comput Sci.

[6] Bauer MJ, Cox AJ, Rosone G, Sciortino M. Lightweight LCP construction for next-generation sequencing datasets. In: Proceedings of WABI. Berlin: Springer; 2012. p. 326–37.

[7] Beller T, Gog S, Ohlebusch E, Schnattinger T (2013). Computing the longest common prefix array based on the Burrows–Wheeler transform. J Discret Algorithms.

[8] Beller T, Zwerger M, Gog S, Ohlebusch E (2013). Space-efficient construction of the Burrows–Wheeler transform. Proc SPIRE.

[9] Bingmann T, Eberle A, Sanders P (2015). Engineering parallel string sorting. Algorithmica.

[10] Bingmann T, Fischer J, Osipov V (2016). Inducing suffix and LCP arrays in external memory. ACM J Exp Algorithmics.

[11] Bingmann T. eSAIS. https://tbingmann.de/2012/esais. Accessed Jun 2017.

[12] Burrows M, Wheeler DJ. A block-sorting lossless data compression algorithm. Digital SRC Research Report. 1994. http://citeseerx.ist.psu.edu/viewdoc/summary?doi=10.1.1.121.6177

[13] Cox AJ, Garofalo F, Rosone G, Sciortino M (2016). Lightweight LCP construction for very large collections of strings. J Discret Algorithms.

[14] Crauser A, Ferragina P (2002). A theoretical and experimental study on the construction of suffix arrays in external memory. Algorithmica.

[15] Crochemore M, Grossi R, Kärkkäinen J, Landau GM. A constant-space comparison-based algorithm for computing the Burrows–Wheeler transform. In: Proceedings of CPM. Berlin: Springer; 2013. p. 74–82.

[16] Dementiev R, Kärkkäinen J, Mehnert J, Sanders P (2008). Better external memory suffix array construction. ACM J Exp Algorithmics.

[17] Dhaliwal J, Puglisi SJ, Turpin A. Trends in suffix sorting: a survey of low memory algorithms. In: Proceedings of ACSC. Canberra: ACSC; 2012. p. 91–8.

[18] Ferragina P, Gagie T, Manzini G (2012). Lightweight data indexing and compression in external memory. Algorithmica.

[19] Fischer J. Inducing the LCP-array. In: Proceedings of WADS. Ansonia: WADS; 2011. p. 374–85.

[20] Flick P, Aluru S. Parallel distributed memory construction of suffix and longest common prefix arrays. In: Proceedings of SC. Carolina: SC; 2015. p. 16:1–10.

[21] Gog S, Moffat A, Culpepper JS, Turpin A, Wirth A (2014). Large-scale pattern search using reduced-space on-disk suffix arrays. IEEE Trans Knowl Data Eng.

[22] Gog S, Ohlebusch E. Fast and lightweight LCP-array construction algorithms. In: Proceedings of ALENEX. Barcelona: ALENEX; 2011. p. 25–34.

[23] Gonnet GH, Baeza-Yates RA, Snider T (1992). New indices for text: pat trees and pat arrays. In: information retrieval.

[24] Gusfield D (1997). Algorithms on strings, trees, and sequences: computer science and computational biology.

[25] Kärkkäinen J, Kempa D. Engineering a lightweight external memory suffix array construction algorithm. In: Proceedings of ICABD. 2014. p. 53–60.

[26] Kärkkäinen J, Kempa D (2016). LCP array construction in external memory. ACM J Exp Algorithmics.

[27] Kärkkäinen J, Kempa D, Puglisi SJ. Parallel external memory suffix sorting. In: Proceedings of CPM. 2015. p. 329–42.

[28] Kärkkäinen J, Kempa D. SAscan. https://www.cs.helsinki.fi/group/pads/SAscan.html. Accessed Jun 2017.

[29] Kärkkäinen, J., Manzini, G., Puglisi, S.J.: Permuted longest-common-prefix array. In: Proceedings of CPM. 2009. p. 181–92.

[30] Kärkkäinen J, Sanders P, Burkhardt S (2006). Linear work suffix array construction. ACM J.

[31] Kasai T, Lee G, Arimura H, Arikawa S, Park K. Linear-time longest-common-prefix computation in suffix arrays and its applications. In: Proceedings of CPM. 2001. p. 181–92.

[32] Knuth DE (1998). The art of computer programming. Sorting and searching.

[33] Ko P, Aluru S (2005). Space efficient linear time construction of suffix arrays. J Discret Algorithms.

[34] Liu WJ, Nong G, Chan WH, Wu Y. Induced sorting suffixes in external memory with better design and less space. In: Proceedings of SPIRE. Bengaluru: SPIRE; 2015. p. 83–94.

[35] Louza FA, Gog S, Telles GP (2017). Optimal suffix sorting and LCP array construction for constant alphabets. Inf Process Lett.

[36] Louza FA, Gog S, Telles GP (2017). Inducing enhanced suffix arrays for string collections. Theor Comput Sci.

[37] Louza FA, Gagie G, Telles GP (2017). Burrows–Wheeler transform and LCP array construction in constant space. J Discret Algorithms.

[38] Louza FA, Telles GP, Ciferri CDA. External memory generalized suffix and LCP arrays construction. In: Proceedings of CPM. 2013. p. 201–10.

[39] Mäkinen V, Belazzougui D, Cunial F (2015). Genome-scale algorithm design.

[40] Manber U, Myers EW (1993). Suffix arrays: a new method for on-line string searches. SIAM J Comput.

[41] Manzini G. Two space saving tricks for linear time LCP array computation. In: Proceedings of SWAT. 2004. p. 372–83.

[42] Munro JI, Navarro G, Nekrich Y. Space-efficient construction of compressed indexes in deterministic linear time. In: Proceedings of SODA. 2017. p. 408–24.

[43] Ng W, Kakehi K (2008). Merging string sequences by longest common prefixes. Inf Process Soc Jpn Digit Cour.

[44] Nong G, Chan WH, Hu SQ, Wu Y (2015). Induced sorting suffixes in external memory. ACM Trans Inf Syst.

[45] Nong G, Chan WH, Zhang S, Guan XF (2014). Suffix array construction in external memory using d-critical substrings. ACM Trans Inf Syst.

[46] Nong G, Zhang S, Chan WH (2011). Two efficient algorithms for linear time suffix array construction. IEEE Trans Comput.

[47] Ohlebusch E (2013). Bioinformatics Algorithms: Sequence Analysis, Genome Rearrangements, and Phylogenetic Reconstruction.

[48] Okanohara D, Sadakane K. A linear-time Burrows–Wheeler transform using induced sorting. In: Proceedings of SPIRE. 2009. p. 90–101.

[49] Puglisi SJ, Smyth WF, Turpin AH (2007). A taxonomy of suffix array construction algorithms. ACM Comput Surv.

[50] Sinha R, Puglisi SJ, Moffat A, Turpin A. Improving suffix array locality for fast pattern matching on disk. In: Proceedings of SIGMOD. 2008. p. 661–72.

